# Performance of a Handheld MicroNIR Instrument for Determining Protein Levels in Sorghum Grain Samples

**DOI:** 10.3390/foods12163101

**Published:** 2023-08-18

**Authors:** Kamaranga H. S. Peiris, Scott R. Bean, Xiaorong Wu, Sarah A. Sexton-Bowser, Tesfaye Tesso

**Affiliations:** 1 Grain Quality and Structure Research Unit, Center for Grain and Animal Health Research, USDA-ARS, Manhattan, KS 66502, USA; shantha.peiris@usda.gov (K.H.S.P.); shawn.wu@usda.gov (X.W.); 2 Department of Agronomy, Kansas State University, Manhattan, KS 66506, USA; sarahann@ksu.edu (S.A.S.-B.); ttesso@ksu.edu (T.T.)

**Keywords:** sorghum, protein, NIR spectroscopy, handheld spectrometer, instrument comparison

## Abstract

Near infrared (NIR) spectroscopy is widely used for evaluating quality traits of cereal grains. For evaluating protein content of intact sorghum grains, parallel NIR calibrations were developed using an established benchtop instrumentation (Perten DA-7250) as a baseline to test the efficacy of an adaptive handheld instrument (VIAVI MicroNIR OnSite-W). Spectra were collected from 59 grain samples using both instruments at the same time. Cross-validated calibration models were validated with 33 test samples. The selected calibration model for DA-7250 with a coefficient of determination (R^2^) = 0.98 and a root mean square error of cross validation (RMSECV) = 0.41% predicted the protein content of a test set with R^2^ = 0.94, root mean square error of prediction (RMSEP) = 0.52% with a ratio of performance to deviation (RPD) of 4.13. The selected model for the MicroNIR with R^2^ = 0.95 and RMSECV = 0.62% predicted the protein content of the test set with R^2^ = 0.87, RMSEP = 0.76% with an RPD of 2.74. In comparison, the performance of the DA-7250 was better than the MicroNIR, however, the performance of the MicroNIR was also acceptable for screening intact sorghum grain protein levels. Therefore, the MicroNIR instrument may be used as a potential tool for screening sorghum samples where benchtop instruments are not appropriate such as for screening samples in the field or as a less expensive option compared with benchtop instruments.

## 1. Introduction

Sorghum (*Sorghum bicolor* (L.) Moench) is the fifth most widely cultivated cereal grain in the world after maize, wheat, rice, and barley and fourth after maize, wheat, and rice in the USA (FAO STAT http://faostat.fao.org (Accessed on 2 August 2022)). In certain regions of the world, sorghum is used mostly for human food. In the USA, sorghum is primarily used as an animal feed ingredient and as a feedstock for biofuel production [1]. In addition, due to the health promoting nature of the crop and its consumer valued qualities, such as being gluten free and a non-GMO product, sorghum’s use as human food is also increasing. Due to the various uses of this crop [2,3], the grain quality requirements for specific uses may vary. Therefore, variety development selection needs to consider grain quality requirements for specific uses. To achieve this efficiently and to streamline the new cultivar development process, it is necessary to integrate high-throughput grain phenotyping technologies into breeding programs [4,5].

Near infrared (NIR) spectroscopy is used as an important analytical tool to evaluate diverse materials for various applications including food, pharmaceuticals, textiles, polymers, etc. [6,7]. NIR spectroscopy methods have been developed for quantitative analysis of numerous traits on intact cereal grains [8,9,10,11,12,13]. The development of NIR spectroscopic methods extends to rapid compositional analyses of grains such as protein [14], starch and amylose [15,16], and phenolics [17].

Plant breeding and germplasm evaluation may require the screening of diverse genotypes and selection to develop new cultivars for specific uses. Screening germplasm for grain quality traits can improve selection of elite lines with improved end-use quality and value. High throughput grain quality analysis by NIR spectroscopic methods is a common method for evaluating grain composition and is typically undertaken in the laboratory using bench-top instruments after the harvesting and processing of the grain samples [14,15,16,17]. A potential bottleneck in screening grain composition by NIR is the need to harvest, clean and transport grain to the laboratory, increasing the cost of time and labor to obtain field phenotypes, delaying availability of quality phenotypes to inform the next nursery season. However, if grain quality traits can be screened in field, either directly on the panicle or after an in-field hand harvest and threshing, potential candidate lines may be rapidly identified. This rapid in-field season test could serve to identify the most promising genotypes for subsequent laboratory analysis. This prioritization of genotypes for subsequent screening will ultimately reduce the overall costs of labor, material, transportation, storage, and sample processing costs for quality trait phenotyping.

Handheld MicroNIR spectrometer devices are available for various NIR spectroscopy applications [18,19,20] and have been used for grain sorghum evaluation. Kosmowsky and Worku [21] investigated the suitability of a miniaturized NIR spectrometer for cultivar identification of barley, chickpea, and sorghum. However, there are no studies that report on the efficacy of handheld NIR devices for the quality trait evaluation of grain sorghum in comparison with the standard method of lab grade benchtop spectrometers. The assessment of tradeoffs for in-field phenotyping verses the precision of current benchtop methods requires a controlled comparison.

Research comparing the use of handheld NIR instruments to benchtop instruments for other products, including forage, coriander seed, lamb, whey, and medicinal plants for various traits has been published [22,23,24,25,26,27,28]. These papers have shown that handheld NIR instruments can provide various degrees of performance compared with benchtop NIR instruments. Because performance of handheld instruments across different products has shown variation in performance, this project was designed to compare a handheld NIR to a benchtop NIR specifically for analysis of intact sorghum grain.

If handheld instruments can be used to assess grain quality traits in-field, then grain quality parameters can be included as selection criteria, thus enhancing the overall breeding efficiency for quality traits. Handheld instruments are less expensive than benchtop instruments, providing a reduced cost of entry for plant breeding programs to perform grain quality phenotyping. However, before attempting to use such handheld micro spectrometers in the field, the feasibility of using such instruments for the evaluation of grain traits must be assessed in comparison with laboratory grade instruments. Therefore, this study was conducted to compare the performance of a handheld MicroNIR instrument with a standard benchtop laboratory NIR spectrometer for evaluation of protein content of sorghum grains.

## 2. Materials and Methods

Grain Samples: Grain samples for the calibration set were selected from two sample sets harvested in 2021. Sample set 1 consisted of 414 grain samples of test market hybrids from 2 private seed companies (3 hybrids), and 3 public breeding programs (23 hybrids) grown under different locations at two dryland sites in Assaria, KS, and Sunray, TX, and an irrigated site in Colby, KS. Sample set 2 consisted of 1864 grain samples from a breeding population entailing breeding lines from a single breeding program grown in Manhattan, KS. Harvested grain samples were cleaned, and the protein contents were determined using an updated in-house NIR protein calibration (R^2^ = 0.92, RMSECV = 0.45%, Slope = 0.93) [29]. The purpose of this preliminary NIR analysis of larger sample sets was to select a smaller set of grain samples with a range of protein content in order to develop a calibration using the two instruments so as to enable a comparison of their performance. A total of 59 grain samples (32 from set 1 and 27 from set 2, about 40 g each) were selected based on the NIR predicted protein content and availability of sufficient seed quantities. Likewise, another set of 33 grain samples were selected from two populations harvested in 2022. The first sample set from 2022 was composed of test market hybrids from 2 private seed companies (8 hybrids), and 1 public breeding program (6 hybrids) grown at two dryland sites in Assaria, KS, and Sunray, TX and an irrigated site at Tribune, KS. The second sample set from 2022 consisted of commercial hybrids grown in 7 locations in Texas and 2 locations in Kansas. Growing locations from both years contained a mix of irrigated and dryland conditions. The 33 samples selected from the populations from the 2022 harvest were used to validate the calibration models developed from the 59 samples harvested in 2021.

Instruments: Samples were scanned by a benchtop instrument ‘DA-7250′ (Perten DA-7250 (Perkin Elmer, Waltham, MA, USA)) and by a battery-powered handheld instrument ‘MicroNIR’ (VIAVI MicroNIR OnSite-W (VIAVI Solutions Inc., Santa Rosa, CA, USA)) at the same time. DA-7250 is a laboratory benchtop instrument weighing about 20 kg and captures data from 950–1650 nm in 5 nm intervals. The MicroNIR is a handheld instrument weighing around 250 g and records spectra from 908–1676 nm at 6.2 nm intervals. Both are photo diode array spectrometers using InGaAs detectors and in which samples are scanned in the reflectance mode. As photodiode array instruments, light reflected from the reference block when collecting the reference spectrum or from the sample when collecting the sample spectrum, is collected by optics and then dispersed by a diffraction grating on to a diode array detector, allowing instantaneous spectral data collection. The DA-7250 is equipped with a 256-pixel detector while the MicroNIR has a 128-pixel detector.

Sample Scanning: Cleaned grain samples were placed in a white Teflon cup (60 mm diameter and 10 mm deep). The MicroNIR equipped with a small measurement collar was held so that it touched the grains on the Teflon cup and the spectrum was recorded by pressing the multifunction button. Immediately after scanning, the same sample was scanned by the DA-7250. Excess grains on the Teflon cup were removed by leveling before scanning the respective sample so that the distance from the surface of grains to the collecting optics of the DA-7250 was uniform for all grain samples. Likewise, when the sample touched the collar of the MicroNIR instrument, the distance between the sample and collection optics was the same for that instrument. Both instruments therefore collected spectra in reflectance mode.

Sample processing for lab analysis: After scanning, grain samples were milled using a cyclone mill equipped with a 0.5 mm screen (Udy Corp, Fort Collins, CO, USA). Protein content of the ground samples were determined by nitrogen combustion using a LECO FP-828p (LECO Corporation, St. Joseph, MI, USA) according to AACI approved method 46-30.01 [30] with an N-to-protein factor of 6.25. The moisture contents of the flour samples were determined by the Perten DA7250 instrument using the in-house flour moisture calibration (R^2^ = 0.98, RMSECV = 0.37%, Slope = 0.98). Grain protein levels were determined on a dry-weight basis based on the flour moisture content. For calibrating the instruments, reference protein levels of the grain samples at the time of scanning were determined accordingly by using the NIR-determined moisture content of grain samples using our grain moisture calibration (R^2^ = 0.99, RMSECV = 0.23%, Slope = 0.99) and dry weight basis protein contents were measured by the LECO method.

Analysis of data: Spectral data analysis was conducted using the Unscrambler X software Version 10.5.1 (CAMO Software AS, Oslo, Norway). Collected raw spectra were preprocessed using 4 different methods (multiplicative scatter correction (MSC), extended multiplicative scatter correction (EMSC), Savitzky–Golay second derivative with 15 datapoints (SG2D15), and standard normal variate (SNV)) and 4 different cross-validated models were developed for each instrument. Those models were validated using the validation test set. The best preprocessing method for the calibration model was selected based on the calibration and validation test results.

## 3. Results

### 3.1. Spectra of Samples

**Figure 1:**
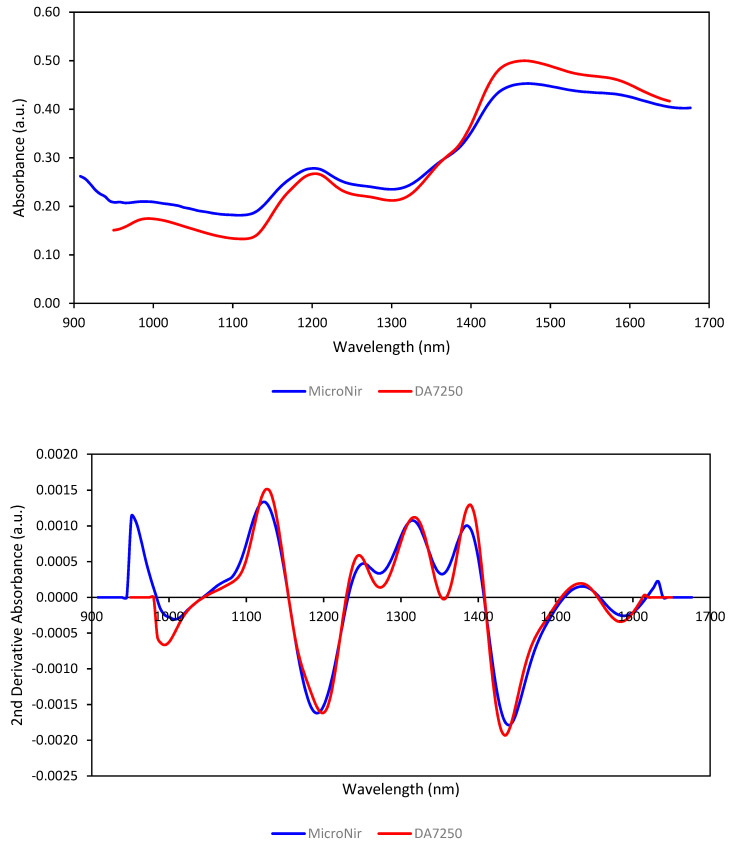
Average raw (above) and second derivative (below) NIR spectra of all 59 samples collected by the two instruments.

The raw spectra collected from all of the grain samples for each instrument and respective second derivative spectra were averaged for comparison and shown in Figure 1. The broad spectral bands in the raw spectra in the spectral range common to both instruments appear similar. The baseline shifts in spectral bands may be due to the differences in the optical configurations of the two instruments. Spectral preprocessing can remove the effects of such baseline shifts as well as scattering effects. The second derivative spectra show that the baseline effects have been minimized.

### 3.2. Grain Protein Diversity in the Sample Population

The protein contents of the 59 samples determined by laboratory nitrogen combustion method as the standard reference method were converted to ‘as is’ protein levels using the moisture content of the grains at the time of scanning. The protein content of the samples ranged from 6.41–16.76% with an average of 10.29% and a standard deviation of 2.70% (Table 1). NIR MC of samples were determined by in-house DA-7250 moisture calibration. The moisture contents of the grain samples ranged from 9.61–11.65% with a mean of 10.54% and a standard deviation of 0.48%. The protein content range of the selected samples represents expected grain protein levels of most sorghum breeding populations.

### 3.3. Protein Calibration Models

**Figure 2:**
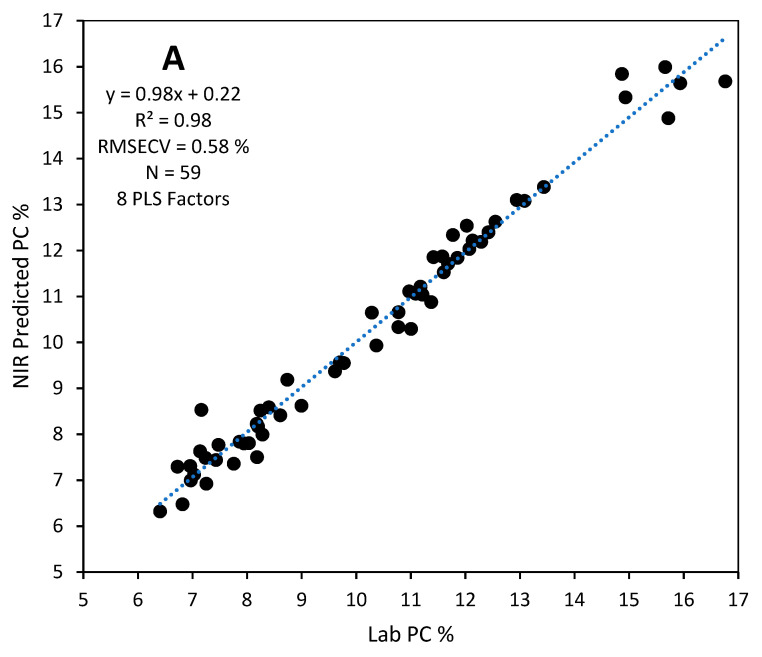

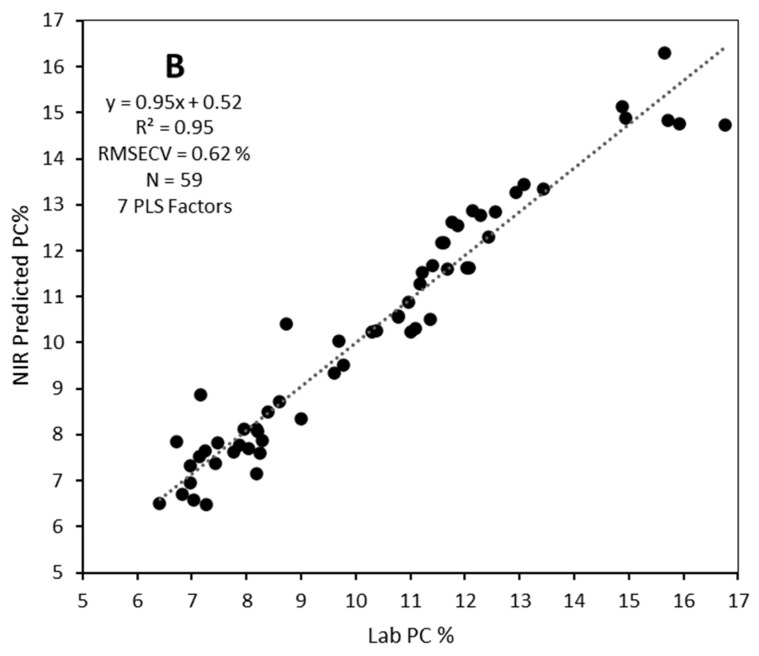
Lab protein content (PC%) vs NIR-predicted PC% of the cross-validated PLS calibration models for (**A**) DA7250 and (**B**) MicroNIR spectrometer.

Calibration models were developed using whole spectral regions for each instrument. Description and performance of protein calibration models developed for the two instruments using four different spectral preprocessing methods and validated with the test set of 33 samples are presented in Table 2. Each calibration model was cross-validated using the leave-one-out cross validation method. The best cross-validated model selected for each instrument (Figure 2) was validated with the test set comprising 33 samples that had been grown in different locations in the following crop year.

Cross-validated calibration models for the MicroNIR had R^2^ ranging from 0.92–0.95, RMSECV ranging from 0.59–0.77% with slope ranging from 0.92–0.95. Respective ranges for the DA-7250 were R^2^ from 0.92–0.98, RMSECV from 0.41–0.76% and slope from 0.94–0.98.

**Figure 3:**
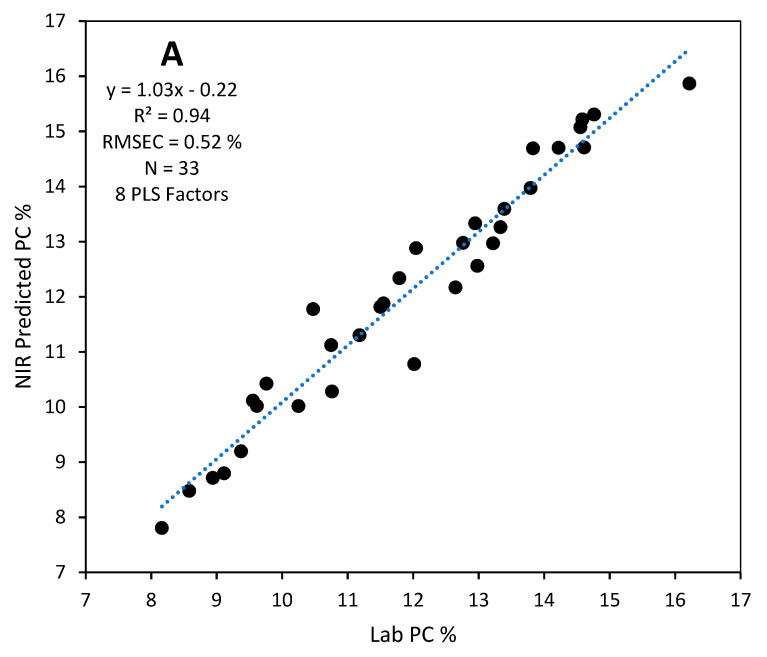

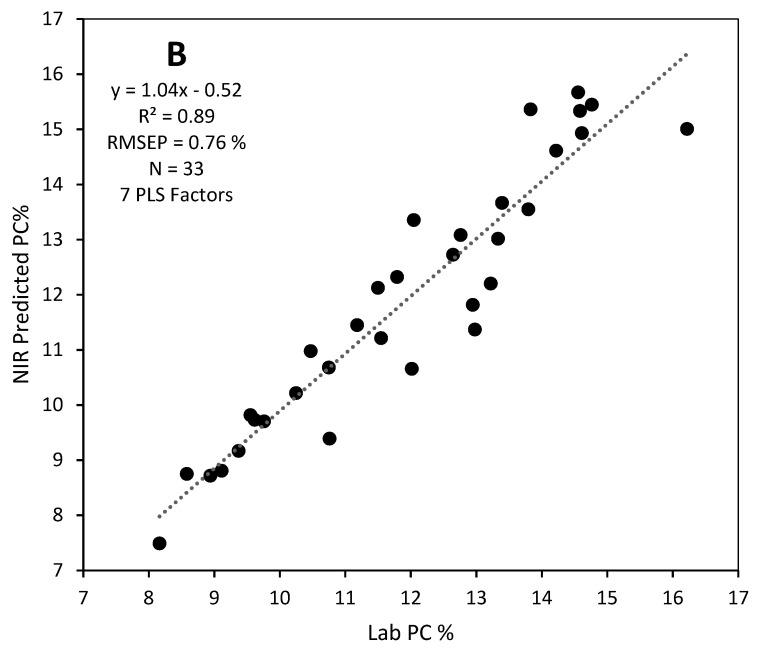
Lab protein content (PC%) vs NIR-predicted PC% of the validation sample set (*n* = 33) for (**A**) DA7250 and (**B**) MicroNIR spectrometer calibration models.

When calibration models were validated with the test set, calibration models constructed with EMSC preprocessed spectra performed better than other models for the MicroNIR instrument while the MSC preprocessed model was the best for the DA7250 (Table 2). The MSC preprocessed model with 8 PLS factors for the DA-7250 (Figure 2A) predicted the protein content of the test set with R^2^ = 0.94, RMSEP = 0.52%, slope = 1.03, Bias = 0.14% having an RPD value of 4.13 (Figure 3A). The EMSC preprocessed MicroNIR calibration model with 7 PLS factors (Figure 2B) predicted the protein content of test set with R^2^ = 0.87, RMSEP = 0.76%, slope = 1.04, Bias = −0.02% and an RPD value of 2.74 (Figure 3B). These prediction performances show that both instruments are suitable for screening protein levels of sorghum grains from plant breeder samples.

**Figure 4:**
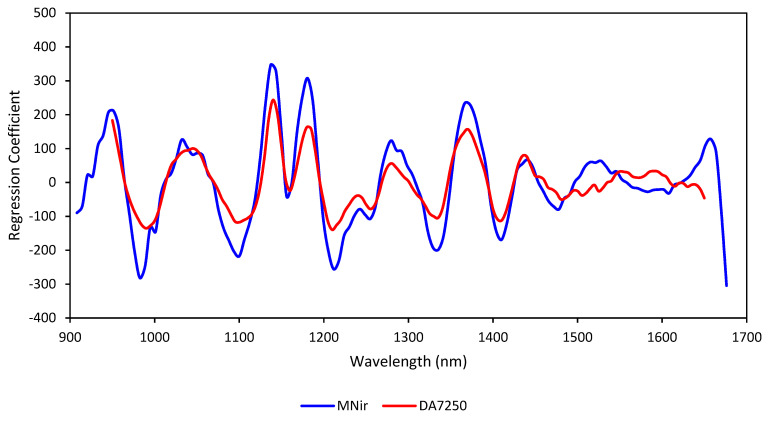
Regression coefficients of cross-validated partial least squares models constructed with the extended multiplicative scatter correction preprocessing method for the MicroNIR and DA7250 instruments.

The regression coefficient plots for the two selected cross validated models for the two instruments are presented in Figure 4. Major regression coefficient peaks are similar for both models. The major positive peaks around 1140, 1180 and 1372 nm are related to protein absorptions while the negative peaks around 985 and 1335 nm are related to starch absorptions while those around 1100, 1155 and 1410 nm to water absorptions [14,31,32]. Therefore, these NIR wavelengths contribute more to the estimation of protein levels in the grain samples by the selected models. Therefore, both calibrations take into account the protein content of grains in the same manner at the above wavelengths related to the NIR absorptions by protein, starch, and moisture.

There is a slight difference in instruments in terms of wavelength range and data collection intervals. The DA-7250 collects data from 950–1650 nm in 5 nm intervals (141 data points) while the MicroNIR records spectra from 908–1676 nm at 6.2 nm intervals (125 data points). Therefore, the MicroNIR instrument used in this study has an advantage in collecting spectra in a wider range than the DA7250, however, DA-7250 collects more spectral data of samples due to its lower data collection interval. Because the most important wavelengths for protein estimation in the calibration model lie in the middle of this spectral range—a range that is common to both instruments—there is no specific advantage of the MicroNIR that derives from these differences in wavelength range. However, if there is a different trait with very important NIR band(s) in the 908–950 nm and 1650–1676 nm ranges, then the MicroNIR instrument used here may have an advantage over the DA-7250 for estimation of that particular trait. Therefore, the results of the comparison of the two instruments may be influenced by the trait being measured as well.

For the protein calibration to offer better performance with a wide variety of samples from different locations, years, moisture levels, breeding programs etc., it needs continuous improvement to enhance its robustness. Therefore, as the instruments are used for future sample sets, a smaller set of subsamples should be validated with reference laboratory analysis and the continual addition of more samples, especially those that show higher prediction errors due to sample matrix variations in the new samples, in order to introduce the expected future sample matrix variations to the calibration model.

The calibration and validation tests of two calibration models developed for two instruments showed that the handheld instrument can be used for the screening of protein levels of grain sorghum. Therefore, there is potential for this instrument to be used to assess the protein content and other grain quality traits of sorghum while the crop is still in the field to inform the breeder when making their final pre-harvest field selection. However, because this protein calibration has been developed with harvested and processed grain samples it should be updated with fresh field sample observations.

Sorghum grain is classified as a “naked caryopsis” and develops without any covering [33] while exposed to a wide range of changing environmental conditions (sunlight, rain etc.), pests and diseases during its development and dry down. Deterioration of the grains exposed to adverse environmental conditions due to chemical, enzymatic, bacterial, fungal and insect activity is referred to as weathering [34]. Weathered sorghum showed changes in the chemical and physical properties of grains [34,35,36]. Such changes in grain properties may result in a wider variation in grain spectra and, in this context, EMSC works best as a pre-processing method of choice for grain sorghum calibrations [29].

Additionally, given that sorghum grain develops as a naked caryopsis on a panicle at the top of the plant [33] this opens the possibility that sorghum grain could be scanned in the field directly on the panicle. However, panicle architecture can vary widely from very compact to very loose in genetically diverse sorghum populations and therefore additional research is needed to determine if grains can be successfully scanned in the field across a variety of sorghum germplasms. It is likely that, for very open panicles, it may not be possible to scan grain directly on the plant. Research is in progress to determine the feasibility of directly scanning sorghum on the plant as well as the suitability of thrashing grain in the field for scanning. Scanning sorghum directly in the field may also require moisture calibrations to be developed with an extended range compared with samples that are harvested, dried, and sent to laboratories for analysis as moisture levels of the grain influence the prediction accuracy of the NIR calibrations [14,37,38].

The calibration model tested in this project could be used to measure PC in grain samples in the 9.61–11.65% range. Whenever samples with predicted protein levels outside this range are found, these can be retested by nitrogen combustion while those outside the range can be incorporated to the calibration to extend its robustness and predictable protein range.

The results of this study demonstrate that a MicroNIR instrument can collect high quality spectra of grains comparable to that of the benchtop instrument and that such spectra may be used for the development of calibrations for grain protein content. With respect to scanning samples, the DA-7250 requires that grain samples be presented in the sample cup on the sample platform. However, because MicroNIR instruments are small units and samples can be presented in different ways, such as grains on a cup or grains on the inflorescence of a plant, MicroNIR instruments can thus easily be adopted for use both under the field and laboratory environments. Therefore, in addition to the possibility of screening grain traits in the field, spectra of other plant parts such as stems, or leaves could also be examined to determine specific traits. For example, studies are currently underway to develop a rapid method for the determination of the dhurrin content of sorghum leaves.

## 4. Conclusions

Inspection of the typical raw spectra and second derivative spectra showed that both instruments can collect spectra of sorghum grain samples with similar spectral quality. NIR protein calibration model performance was influenced by the method used to preprocess data. The interaction between data pre-processing techniques and calibration performance in this study suggests this should be evaluated in future work with miniaturized NIR instruments. Models developed with EMSC preprocessed spectra performed better for the tested MicroNIR instrument while the MSC preprocessing was best for the DA7250 instrument. The benchtop DA-7250 performed better than the handheld instrument, however, the handheld instrument was able to predict the protein levels of grain samples with an RMSEP of 0.76% compared with that of 0.52% for the DA-7250. Therefore, the handheld instrument tested here may be used as an alternative low-cost instrument. Due to its smaller size and versatile sample presentation, handheld NIR instruments in general have the potential to be used for in-field phenotyping of sorghum grains and or other plant parts. As this research was conducted in a controlled laboratory environment, this project provides the foundation for future research using miniaturized NIR instruments in both laboratory and field conditions for the analysis of sorghum grain.

## Figures and Tables

**Table 1:** Descriptive statistics of protein content of grain samples.

Sample Set	N *	Min	Max	Avg	STD
Calibration set	59	6.41	16.76	10.29	2.70
Validation set	33	8.16	16.22	11.92	2.11

* N: Number of samples; Min: minimum; Max: maximum; Avg: average; STD: standard deviation.

**Table 2:** Calibration and validation statistics of partial least squares models constructed with different preprocessing methods for the two instruments.

		Calibration Model					Validation Test					
Instrument	Preprocessing	LV	N	R^2^	RMSECV	Slope	N	R^2^	RMSEP	Slope	Bias	RDP
MicroNIR	MSC	9	59	0.95	0.59	0.95	33	0.84	0.82	1.07	0.29	2.70
	EMSC	7	59	0.95	0.62	0.95	33	0.87	0.76	1.04	−0.02	2.74
	SG2D15	6	59	0.92	0.77	0.92	33	0.69	1.16	1.00	−0.38	1.90
	SNV	9	59	0.95	0.61	0.95	33	0.80	0.92	1.08	0.42	2.70
DA7250	MSC	8	59	0.98	0.41	0.98	33	0.94	0.52	1.03	0.14	4.13
	EMSC	6	59	0.97	0.49	0.97	33	0.80	0.92	1.03	0.46	2.57
	SG2D15	5	59	0.92	0.76	0.94	33	0.78	0.97	1.02	−0.36	2.32
	SNV	8	59	0.98	0.42	0.98	33	0.80	0.94	0.93	−0.21	3.46

MSC: multiplicative scatter correction; EMSC: extended multiplicative scatter correction; SG2D15: Savitzky–Golay second derivative with 15 data points; SNV: standard normal variate; LV: number of latent variables (PLS Factors); N: number of samples; R^2^: coefficient of determination; RMSECV: root mean square error of cross validation; RMSEP: root mean square error of prediction; RPD: ratio of performance to deviation.

## Data Availability

The data used to support the findings of this study are available at https://data.nal.usda.gov/.
